# HBV Infection-Related *PDZK1* Plays an Oncogenic Role by Regulating the PI3K-Akt Pathway and Fatty Acid Metabolism and Enhances Immunosuppression

**DOI:** 10.1155/2022/8785567

**Published:** 2022-08-23

**Authors:** Xin Chen, Xiaodong Wang, Feng Zhu, Chao Qian, Fanggui Xu, Xin Huang, Wenjie Zhang, Beicheng Sun

**Affiliations:** ^1^Department of Hepatobilliary Surgery of Drum Tower Clinical Medical College, Nanjing Medical University, Nanjing, 210008 Jiangsu Province, China; ^2^Department of General Surgery, Sir Run Run Hospital, Nanjing Medical University, Nanjing, 211100 Jiangsu Province, China; ^3^Department of General Surgery, Northern Jiangsu People's Hospital, Yangzhou, 225001 Jiangsu Province, China; ^4^Department of Hepatobilliary Surgery, Nanjing Drum Tower Hospital Clinical College of Traditional Chinese and Western Medicine, Nanjing University of Chinese Medicine, China

## Abstract

**Methods:**

We compared differentially expressed genes (DGEs) in HBV-positive and -negative tumor samples and conducted a Spearman correlation study between the DGEs and HBV titers within The Cancer Genome Atlas (TCGA). Moreover, we validated the results of our in-house samples.

**Results:**

In this study, we discovered a series of genes that correlated statistically with HBV infection based on the TCGA database. These genes were related to increased inflammation and some oncogenic signaling pathways via Gene Set Enrichment Analysis (GSEA). *PDZK1* is an ideal gene, which mostly relates positively to HBV infection; moreover, it is overexpressed in human HCC, especially in those HBV-infected HCCs. After analyzing the TCGA data and performing a verification study using our own samples, *PDZK1* expression was investigated to be significantly associated with PI3K-Akt signaling and fatty acid metabolism. Further, single-sample GSEA analysis of tumor immune cell infiltration gene sets revealed that high *PDZK1*expression in HCC tissues was significantly associated with increased tumor-associated macrophages (TAMs) and regulatory T cells(Tregs).

**Conclusions:**

*PDZK1* is an HBV infection-related gene, which plays oncogenic roles, possibly due to enhancing PI3K-Akt, fatty acid usage in tumor cells and TAMs, and Treg-induced immunosuppression.

## 1. Introduction

Chronic hepatitis B virus (HBV) infection is the leading cause of hepatocellular carcinoma (HCC) globally [1]. Chronic carriers have a more than 100-fold increased relative risk of tumor development. Several mechanisms have been proposed to induce HCC using HBV [2]. HBV causes 80% of liver cancer cases and is the second most common carcinogen after smoking. Babies born to e-positive mothers have approximately a 90% risk of becoming persistent carriers after perinatal infection, and preschoolers have a 30% risk [2, 3]. Only 5% to 10% of adults become persistent carriers after infection. In individuals persistently infected with HBV, 10%-30% will develop cirrhosis and HCC. In persistently infected individuals, these highly different results in clearance rates and disease outcomes cannot be fully explained by differences in immune, viral, or environmental factors. Therefore, differences in host genetic factors may influence the natural history of hepatitis B.

The genetic factors that affect the outcome of HBV infection include human leukocyte antigen (HLA), cytokines, chemokines, mannose-binding proteins, vitamin D receptors, and Toll-like receptors (TLRs) [4–8], such as HLA-DPA1 rs3077 and HLA-DPB1 single nucleotide polymorphisms (SNPs) [9] and TNF-alpha promoter SNP and/or haplotypes [10]. However, it is difficult to verify other genes in different populations. Moreover, a single gene or SNP cannot fully explain disease susceptibility because human traits are inherited from multiple genes. The main advantage of genetic association analysis is that it uses distortions in the genetic frequency distribution of comparative populations to detect disease-related genes. However, this advantage has potential drawbacks that can lead to false-positive associations and the deletion of important loci. False-positive associations may result from sampling errors in substructured study populations, variations associated with multiple statistical tests, and linkage imbalances between labeled SNPs and actual disease-related SNPs. Since almost all investigations into HBV-associated HCC have been based on epidemiology and the detailed molecular mechanisms remain unexplored, we can predict whether HBV infection is present in liver cancer samples from the TCGA database based on a report published in *Nature Communications* [11].

In this study, we compared differentially expressed genes (DGEs) in HBV-positive and -negative tumor samples according to previous reports and conducted a Spearman correlation study between the DGEs and HBV titers within TCGA. We found that *PDZK1* may be an ideal gene that is associated with HBV infection and is significantly differentially expressed in cancer and noncancer tissues. Below, we discuss the potential effects of *PDZK1* on gene signaling pathways and the tumor microenvironment.

## 2. Materials and Methods

### 2.1. Study Subjects

The hospital-based case-control study consisted of 80 study subjects newly diagnosed with HCC, with 50 cases being HBV positive and 30 cases being HBV negative. All subjects were recruited from Sir Run Run Hospital, Nanjing Medical University, and Northern Jiangsu People's Hospital between July 2016 and June 2020. Study subjects with other hematological disorders, previous history of cancer, radiotherapy, immunosuppression/antiviral medication, and chemotherapy were excluded. The cancer-free control subjects from the same geographic area showed no genetic relationship with the cases. Study subjects were classified according to the World Health Organization classification. The Institutional Review Board of Sir Run Run Hospital, Nanjing Medical University, and Northern Jiangsu People's Hospital approved this study, and every patient gave written informed consent.

### 2.2. Bioinformatics Analysis

Liver hepatocellular carcinoma (LIHC) liver cancer data from the TCGA database were used to extract samples with known HBV infection status according to previous reports. The difference between the two groups of transcripts was calculated using the R software package Limma (v.3.40.6), and the absolute value of fold change (Fc) abs(FC) > 0.5. Adjusted *P* < 0.05 was used to identify DGEs. clusterProfiler (v.3.12.0) and PHEATMAP (v.1.0.12) were used for cluster analysis and heat map generation Gene Ontology (GO), respectively, and Kyoto Encyclopedia of Genes and Genomes (KEGG) pathway analysis using Cluego25 (v.2.5.5). Data from the TCGA-liver hepatocellular carcinoma (LIHC) dataset were used to extract samples with known HBV infection status according to previous reports. The critical value was a false detection rate < 0.05.

### 2.3. The Prognosis of Dysregulated Genes Was Analyzed Using Gene Expression Profiling Interactive Analysis (GEPIA) with the Kaplan–Meier Plotter

GEPIA (http://gepia.cancer-pku.cn/) [12] is a new web-based tool containing sequencing expression data from 9736 tumor samples of 33 cancer types and 8587 normal samples. The database includes various analysis modules, such as differential gene expression, survival and prognosis, correlation, and dimensionality reduction analyses.

#### 2.3.1. Real-Time Polymerase Chain Reaction (PCR)

Total RNA in HCC tissues was isolated using the TRIzol reagent. The expression of genes of interest was detected using the cyber-green-based real-time PCR. The primers for genes used in the study are listed in Table 1.

#### 2.3.2. Immunohistochemistry Staining

Sections were stained according to the previous publication. The sections were incubated with primary mouse antihuman Ab for CD68(ab213363), CD163(ab182422), CD4(ab183685), ITGA6 (ab181551), RAF1 (ab181115), FGFR3 (ab133644), ADH5(ab177932), FASN (ab128870), and Foxp3 (ab215206). The sections were stained with 3,3′-diaminobenzidine according to the manufacturer's protocols and then mounted and photographed using a digitalized microscope camera (Nikon, Japan).

### 2.4. Statistical Analysis

Data are presented as the mean ± SEM. The *χ*^2^ test and Student's *t*-test analysis of variance were used to evaluate differences in demographic and clinical characteristics. Kaplan-Meier survival curves were generated, and the log-rank test was used to investigate the significance of various variables for survival. All expression experiments conducted *in vitro* were repeated at least thrice with triplicate samples. Pearson's correlation analysis was used to analyze the relationship of associated factors. Statistical analysis was performed using STATA 9.2 and presented with the GraphPad Prism (CA, USA). In all cases, *P* < 0.05 was considered significant.

## 3. Results

### 3.1. HBV Infection-Related Genes Are Associated with Increased Inflammation and Several Oncogenic Signaling Pathways

**Figure 1:**
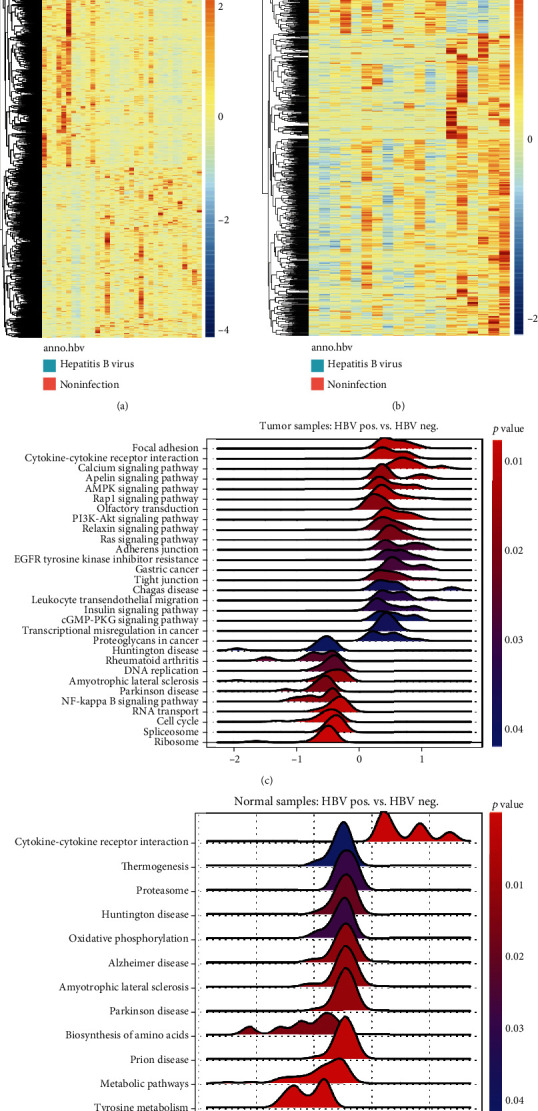
HBV infection-related genes were associated with increased inflammation and several oncogenic signaling pathways. (a, b) Heat map of all significantly differently expression genes (DEGs) (*n* = 964, HBV Pos. vs. HBV Neg, in tumor tissues, and *n* = 1098, HBV Pos. vs. HBV Neg. in normal tissues). (c, d) GSEA results obtained by using DEGs by comparing between HBV Pos. and HBV Neg. Samples in tumor (c) and normal (d) tissues.

Based on previous reports [10], we obtained 11 tumor and 6 nontumor HBV-infected samples as well as 22 tumor and 13 nontumor HBV noninfected samples in the TCGA-LIHC database. First, we compared the differential gene expression between HBV-infected and noninfected samples in normal or tumor tissues and obtained DEGs using the criterion with an absolute value of log Fc > 0.5 and adjusted *P* value of <0.05 (Figures 1(a) and 1(b)). After the DEGs were enriched, GSEA was performed using a typical KEGG pathway subset(CP). The results suggest that HBV infection is associated with an increased inflammatory response due to enrichment of signaling pathways of “leukocyte transendothelial migration” and “cytokine-cytokine receptor interactions” (Figures 1(c) and 1(d)). However, DEGs obtained from tumor tissues were associated with carcinogenic signaling pathways such as focal adhesion, PI3K-Akt, and Ras signaling pathways (Figure 1(c)). These results agreed with previous reports of HBV-induced enhancement of liver inflammation. However, our study also provides new insights that HBV infection in tumor tissues has more oncogenic characteristics than in HBV-negative tumors, which may be mediated by pathways, such as PI3K and Ras signaling pathways.

### 3.2. PDZK1 Is an HBV-Infection-Related Oncogene in Human HCC

**Figure 2:**
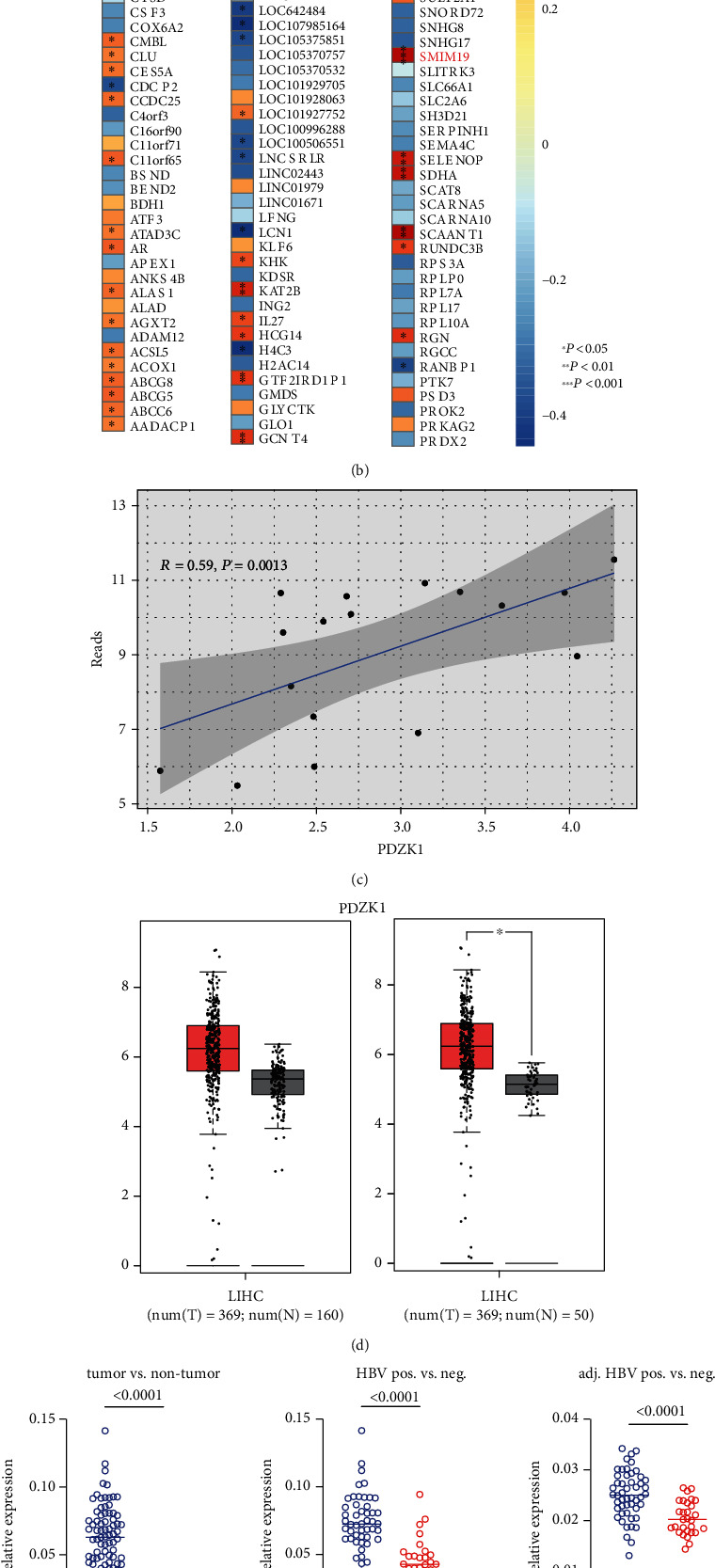
PDZK1 is one of the HBV-infection-related oncogenes in human HCC. (a) Venny map indicating the common up- or downregulated genes both in cancer and normal samples from TCGA-LIHC. (b) Heat map reflating either *R* or *P* value obtained from multiple correlation study between HBV reads and gene expression level of common genes. (c) Scatter plot reflecting the correlation between HBV reads and PDZK1 expression in the TCGA samples. (d) The comparison of PDZK1 expression between normal and tumor samples from TCGA-LIHC or TCGA-LIHC and GTEx database. (e) Real-time PCR detection PDZK1 transcription in the sample sets indicated in the figure. (f) Scatter plot reflecting the correlation between HBV reads and PDZK1 expression from clinical samples.

To find the ideal candidate gene for further study, we found commonly up- or downregulated DEGs between tumor and normal samples using the online tool, http://Venny2.1. As presented in the Venny map (Figure 2(a)), we found 27 upregulated and 82 downregulated common genes. Next, we conducted a multiple correlation study on HBV reads provided by previous reports [11] and the expression of each common gene. We found that 63 genes were significantly linearly correlated with HBV reads, with 49 correlating positively and 14 negatively (Figure 2(b)). Among these genes, we found that *PDZK1* is an ideal oncogene regulated by HBV. We found that *PDZK1* showed a good linear correlation with HBV reads (*R* = 0.59, *P* = 0.0013) (Figure 2(c)), and the data in TCGA-LIHC suggested that this gene was significantly overexpressed in HCC tissues (Figure 2(d)).

To verify these TCGA results, we collected HCC samples with and without HBV infection (*n* = 50 and *n* = 30). First, we confirmed the high expression of the *PDZK1* gene in human hepatocellular carcinoma (Figure 2(e)). Additionally, we found that compared with HBV-negative tissues, *PDZK1* expression increased significantly in HBV-positive tissues, both in tumors and in adjacent tissues (Figure 2(e)). Finally, we studied the linear correlation between *PDZK1* expression and HBV titer, and a good correlation was observed (*R* = 0.9, *P* < 0.0001) (Figure 2(f)).

### 3.3. High *PDZK1* Expression in Human HCC Is Associated with Inflammation, Oncogenic Characteristics, and Fatty Acid Metabolism

**Figure 3:**
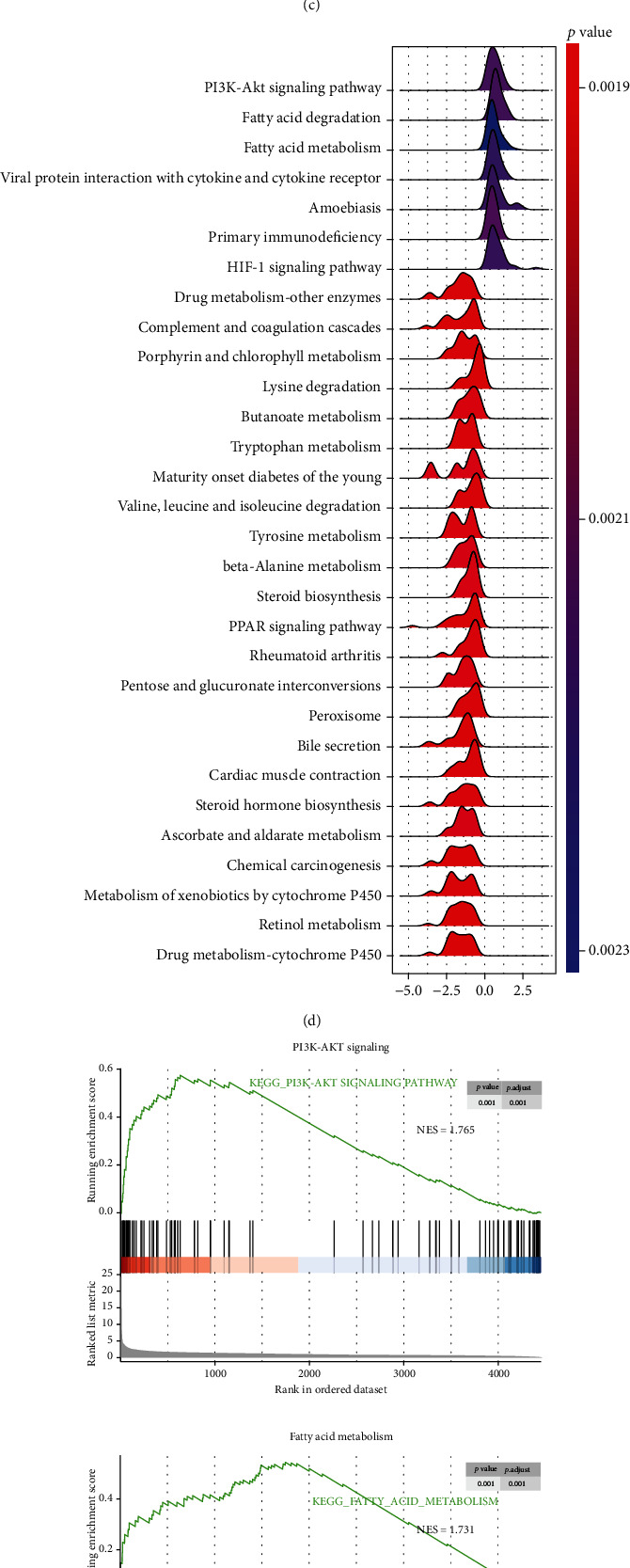
PDZK1 has oncogenic roles by enhancing PI3K/AKT and fatty acid metabolism. (a) Heat map of all DEGs obtained by comparing PDZK1 top 20 high and top 20 low samples. (b) GO enrichment for biological process (BP) based on the DEGs. (c) KEGG enrichment based on the DEGs. (d) GSEA analysis for gene expression fold change between PDZK1 top 20 high and top 20 low samples. (e) GSEA plot for PI3K-AKT, fatty acid metabolism, and fatty acid degradation pathways. (f) Heat map for key genes representing PI3K-AKT, fatty acid metabolism, and fatty acid degradation signaling.

By comparing the 40 samples with the highest and lowest PDZK1 gene expression in TCGA-LIHC, 4453 DEGs (adjusted *P* < 0.05) were obtained, among which 2049 genes were downregulated and 2024 genes were upregulated (Figure 3(a)). Furthermore, DEGs were used for GO and KEGG enrichment. For GO (BP) enrichment, we found that *PDZK1* is associated with increased inflammatory response, reflected in terms such as “neutrophil activation” and “T-cell activation” (Figure 3(b)). For KEGG enrichment, we found that the PI3K-Akt signaling pathway ranked first among all signaling pathways (Figure 3(c)). Next, we performed GSEA analysis on fold change values of all coding genes (25,717 genes), and we found that PI3K-Akt was also the first significant signaling pathway. The second and third signaling pathways of “fatty acid degradation” and “fatty acid metabolism” were found (Figure 3(d)). Normalized enrichment scores (NES) of the three genomes were 1.765, 1.731, and 1.725 (Figure 3(e)). Some core genes belonging to these three gene sets are listed in Figure 3(f).

**Figure 4:**
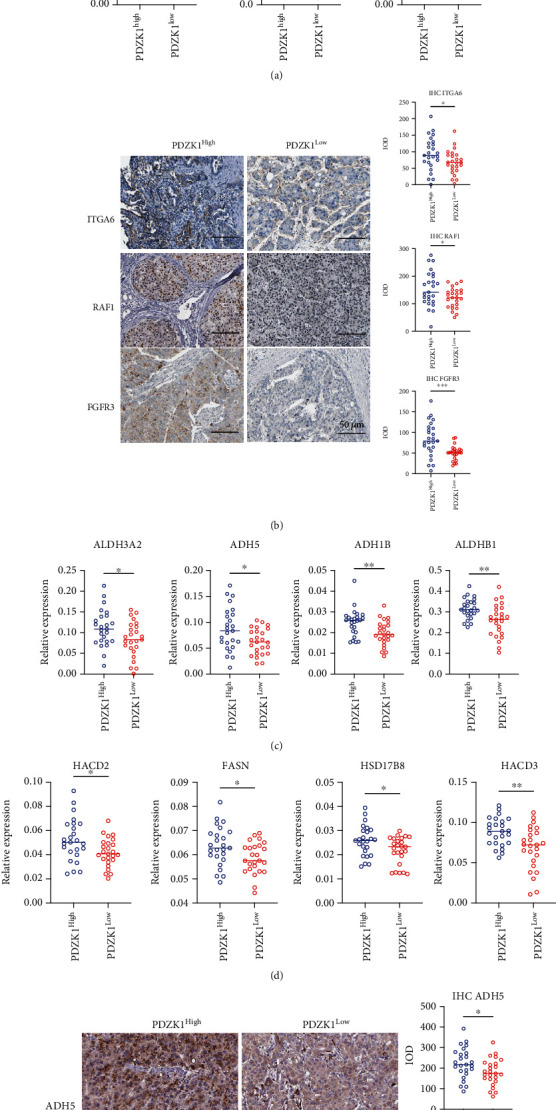
PDZK1 was correlated to increased PI3K/AKT and fatty acid metabolism. (a) Real-time PCR detection PI3K-AKT signaling-related genes indicated in the figure in PDZK1 high and low expression samples. (b) IHC staining of ITGA6, RAF1, and FGFR3 in PDZK1 high and low expression samples. (c, d) Real-time PCR detection fatty acid metabolism and degradation-related genes indicated in the figure in PDZK1 high and low expression samples. (e) IHC staining of ADH5 and FASN in PDZK1 high and low expression samples. Data are presented as mean ± SEM and were analyzed using Student's *t*-test (^∗^*P* < 0.05, ^∗∗^*P* < 0.01; ns: not significant, *P* > 0.05).

Additionally, we used our clinical samples (*PDZK1*^High^, *n* = 25, and *PDZK1*^Low^, *n* = 25) to verify the relationship between *PDZK1* expression and multiple signaling pathways. First, six genes, *MET*, *IL6R*, *FGFR3*, *BCL2L1*, *RAF1*, and *ITGA6*, were selected to be associated with the PI3K-Akt signaling pathway. We found that these six genes were significantly overexpressed in HCC samples with high *PDZK1* expression compared with the HCC samples with low *PDZK1* expression (Figure 4(a)). Simultaneously, the protein expressions of ITGA6, RAF1, and FGFR3 were detected using immunohistochemical staining, and we found that these three proteins were also overexpressed in *PDZK1* high-HCC samples (Figure 4(b)). Next, real-time PCR technology was used to analyze some components of genes belonging to “fatty acid degradation” and “fatty acid metabolism” signal pathways. Fatty acid degradation-related genes included *ALDH3A2*, *ADH5*, *ADH1B*, and *ALDH1B1* and fatty acid metabolism-related genes *HACD2*, *FASN*, *HSD17B8*, and *HACD3*. These genes were relatively high in HCC samples with high *PDZK1* expression (Figures 4(c) and 4(d)). Finally, we used immunohistochemistry to detect the expression of ADH5 and FASN proteins in HCC tissues, suggesting that the expression of these two key enzymes in the metabolic process was high in HCC samples with high *PDZK1* expression (Figure 4(e)).

### 3.4. Expression of *PDZK1* in HCC Tissues Was Associated with Treg and TAM-Induced Immunosuppression

**Figure 5:**
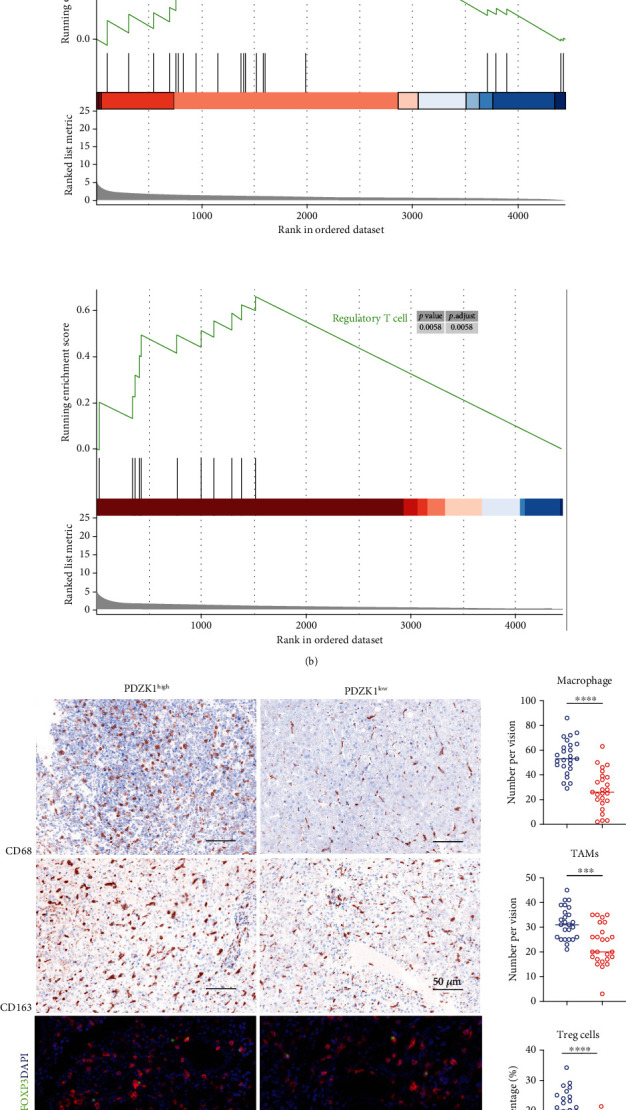
Expression of PDZK1 in HCC tissues was associated with Treg, and tumor-associated macrophages (TAMs) induced immunosuppression. (a) ssGSEA analysis for PDZK1 top 20 high and low expression TCGA samples by using previously reported gene sets. (b) GSEA analysis for PDZK1 top 20 high and low expression TCGA samples. (c) IHC and multiple color IHC staining for CD68, CD163, and CD4/FOXP3 in PDZK1 high and low expression samples. Data are presented as mean ± SEM and were analyzed using Student's *t*-test (^∗^*P* < 0.05, ^∗∗^*P* < 0.01; ns: not significant, *P* > 0.05).

To study the relationship between the *PDZK1* gene and the tumor microenvironment, we used single-sample GSEA to analyze the possible immune cell infiltration in the samples from the TCGA-LIHC database. We found that tissues with high *PDZK1*expression had more abundant infiltration of macrophages and Treg cells (Figure 5(a)). Then, this result was confirmed using GSEA. The results suggested that the DEGs between *PDZK1* high/low comparison were enriched in the genes of macrophages and regulatory T cells (Figure 5(b)). Then, we validated the bioinformatics analysis by staining macrophages and TAMs with IHC staining for CD68 and CD163, and we found a high infiltration of macrophages, especially TAMs, in *PDZK1*-high expression samples (Figure 5(c)). Simultaneously, we found that the proportion of Treg cells in HCC tissues with high *PDZK1* expression was also higher (Figure 5(c)).

## 4. Discussion

Our study found that *PDZK1* is a gene highly linearly correlated with HBV infection and may promote the occurrence and development of liver cancer by promoting the PI3K-Akt pathway and fatty acid metabolism. PDZK1 is an adaptor protein containing four PDZ domains, which binds to the carboxy-terminal cytoplasmic tail of SR-B1 through PDZ domain 1 (PDZ1) and PDZ3, whereas PDZ4 mediates interaction with membrane lipids [13, 14]. It tissue specifically protects SR-B1 from degradation. In the absence of *PDZK1*, SR-B1 protein levels were significantly reduced in the liver (95%) and moderately decreased in the intestinal tissue (50%) but were unaffected in steroids, endothelial cells, or macrophages [14, 15]. SR-B1 and *PDZK1* also play an important role in HDL-mediated signal transduction in vascular cells [16, 17]. Mice deficient in *PDZK1* showed an increase in HDL-C, consistent with the loss of SR-B1 protein in the liver [18, 19]. *PDZK1*/ApoE DKO mice developed greater diet-induced aortic atherosclerosis compared with control ApoE knockout mice when fed a high-fat, Western-induced atherosclerotic diet [20, 21]. From the reports above, we can conclude that *PDZK1*is related positively to fatty acid metabolism in different types of normal cells. However, we first reported that *PDZK1*is related significantly to fatty acid metabolism in human liver cancer.

In this study, we found that increased *PDZK1* in human liver cancer, especially HBV-positive cancers, was related to enhanced P3IK-Akt signaling. However, we found that the reports concerning the relationship between *PDZK1* and PI3K-Akt signaling in different tissues are controversial. In studies of intestinal inflammation, soluble uric acid increased the expression of *PDZK1* and *ABCG2* [22]. Stimulation of soluble uric acid also promoted the transport of *ABCG2* from the intracellular compartment to the plasma membrane and increased its transport activity. TLR4-NLRP3 inflammasome inhibitors or PI3K-Akt signaling inhibitors can partially reduce the upregulation of *PDZK1* and *ABCG2* by soluble uric acid. Additionally, *PDZK1*downregulation significantly inhibited *ABCG2* expression and transport activity. However, it was unaffected by the activation of soluble uric acid, suggesting that *PDZK1* plays a key role in regulating*ABCG2*. These results suggest that uric acid promotes excretion by activating the TLR4-NLRP3 inflammasome and PI3K-Akt signaling pathway to upregulate the expression of *PDZK1* and *ABCG2* in intestinal cells [22]. However, other studies have shown that *PDZK1* can inhibit PI3K-Akt activation. In this study, PTEN was identified as a new *PDZK1* binding protein by binding to the PDZ domain of *PDZK1* through its carboxyl terminal. Through direct interaction with PTEN, *PDZK1* inhibited PTEN phosphorylation at S380/T382/T383, further enhancing the ability of PTEN to inhibit PI3K-Akt activation. *In vivo* and *in vitro* studies have shown that *PDZK1* inhibits PI3K-Akt activation by inhibiting PTEN phosphorylation, thereby inhibiting the proliferation of gastric cancer cells. Frequent downregulation of *PDZK1* expression in gastric cancer specimens is associated with the disease progression and poor prognosis of study subjects with gastric cancer [23]. In clinical specimens, *PDZK1*downregulation is associated with PTEN inactivation, Akt signaling pathway, and cell proliferation activation. Our studies in liver cancer have shown that high expression of *PDZK1* in liver cancer correlates highly with PI3K-Akt pathway activation. This difference in the role of *PDZK1* in different tumors may be related to tissue differences.

Thus far, there are no reports concerning the effect of *PDZK1* on the tumor microenvironment, and we reported for the first time that high expression of *PDZK1* was associated with immunosuppression caused by increased TAMs and Treg cells.

In summary, we found that the *PDZK1* gene correlated highly with HBV infection, and *PDZK1* might play oncogenic roles by relating positively with PI3K-Akt activation, fatty acid metabolism, and immunosuppression in human HCC.

## Figures and Tables

**Table 1:** Primer sequence for genes of interest.

Gene symbol	Sequence	Amplificon (bp)
PDZK1	TTCCTGCGAATTGAGAAGGAC	164
	CCCCGAATCGCATTTAAGTGAA	
MET	AGCAATGGGGAGTGTAAAGAGG	191
	CCCAGTCTTGTACTCAGCAAC	
IL6R	CCCCTCAGCAATGTTGTTTGT	171
	CTCCGGGACTGCTAACTGG	
FGFR3	CCCAAATGGGAGCTGTCTCG	109
	CCCGGTCCTTGTCAATGCC	
BCL2L1	GAGCTGGTGGTTGACTTTCTC	119
	TCCATCTCCGATTCAGTCCCT	
RAF1	GGGAGCTTGGAAGACGATCAG	165
	ACACGGATAGTGTTGCTTGTC	
ITGA6	ATGCACGCGGATCGAGTTT	160
	TTCCTGCTTCGTATTAACATGCT	
ALDH3A2	AAACCAGTTAAGAAGAACGTGCT	103
	CGAAGGGGTAATTCCAAGCTC	
ADH5	ATGGCGAACGAGGTTATCAAG	202
	CATGTCCCAAGATCACTGGAAAA	
ADH1B	CCCGGAGAGCAACTACTGC	224
	AACCAGTCGAGAATCCACAGC	
ALDH1B1	AGGGGGCTGTTTATGGTGG	167
	GGTACGGATACTTTTCCCAGAGT	
HACD2	GCTGCGAAGTGGAAACCATC	135
	CCTCCTTCTGCACACATTTGAA	
FASN	AAGGACCTGTCTAGGTTTGATGC	106
	TGGCTTCATAGGTGACTTCCA	
HSD17B8	CCTGGTGTCCAATGGTTGTC	60
	ACCTTTCCTACGATGCTACTGAT	
HACD3	TGGAGCCCGTGAAAAAGAGC	75
	TCTCCTTCATCTTAGAGGCCAC	

## Data Availability

The data were obtained from the TCGA database.
